# Sub-ppm Formaldehyde Detection by *n*-*n* TiO_2_@SnO_2_ Nanocomposites

**DOI:** 10.3390/s19143182

**Published:** 2019-07-19

**Authors:** Abulkosim Nasriddinov, Marina Rumyantseva, Artem Marikutsa, Alexander Gaskov, Jae-Hyoung Lee, Jae-Hun Kim, Jin-Young Kim, Sang Sub Kim, Hyoun Woo Kim

**Affiliations:** 1 Chemistry Department, Moscow State University, Moscow 119991 Russia; 2 Faculty of Materials Science, Moscow State University, Moscow 119991 Russia; 3 Department of Materials Science and Engineering, Inha University, Incheon 22212, Korea; 4 School of Materials Science and Engineering, Hanyang University, Seoul 04763, Korea

**Keywords:** formaldehyde gas sensor, sub-ppm concentration, UV light, metal-oxide nanocomposites, SnO_2_, TiO_2_

## Abstract

Formaldehyde (HCHO) is an important indicator of indoor air quality and one of the markers for detecting lung cancer. Both medical and air quality applications require the detection of formaldehyde in the sub-ppm range. Nanocomposites SnO_2_/TiO_2_ are promising candidates for HCHO detection, both in dark conditions and under UV illumination. Nanocomposites TiO_2_@SnO_2_ were synthesized by ALD method using nanocrystalline SnO_2_ powder as a substrate for TiO_2_ layer growth. The microstructure and composition of the samples were characterized by ICP-MS, TEM, XRD and Raman spectroscopy methods. The active surface sites were investigated using FTIR and TPR-H_2_ methods. The mechanism of formaldehyde oxidation on the surface of semiconductor oxides was studied by in situ DRIFTS method. The sensor properties of nanocrystalline SnO_2_ and TiO_2_@SnO_2_ nanocomposites toward formaldehyde (0.06–0.6 ppm) were studied by in situ electrical conductivity measurements in dark conditions and under periodic UV illumination at 50–300 °C. Nanocomposites TiO_2_@SnO_2_ exhibit a higher sensor signal than SnO_2_ and a decrease in the optimal measurement temperature by 50 °C. This result is explained based on the model considering the formation of *n*-*n* heterocontact at the SnO_2_/TiO_2_ interface. UV illumination leads to a decrease in sensor response compared with that obtained in dark conditions because of the photodesorption of oxygen involved in the oxidation of formaldehyde.

## 1. Introduction

Formaldehyde, HCHO, a colorless gas with an unpleasant odor, is a toxic compound that causes, in trace concentrations of 0.1–0.5 mg/m^3^, serious diseases of the respiratory tract, gastrointestinal tract and eyes. Biochemical oxidation of HCHO in human body occurs with the formation of carbon dioxide and formic acid, which, with prolonged exposure, causes asthma, pulmonary edema, and cancer. Formaldehyde is widely used in the manufacturing of polymeric materials for flooring, furniture, heat and electrical insulation, artificial tissues, plastic windows, etc. In addition, a concentrated HCHO solution (formalin) is used in medicine for disinfection, and in the food industry for the preservation of fruits and vegetables. Formaldehyde is also included in some cosmetics and personal care products. A detailed description of the characteristics of formaldehyde and its effects on health can be found in Ref. [1]. According to the World Health Organization, the maximum permissible concentration of formaldehyde in the air of the working area is 0.5 mg/m^3^ (0.4 ppm), in the air of the residential area is 0.125 mg/m^3^ (0.1 ppm) [1,2].

Recently, it was proposed that formaldehyde is one of the markers for lung cancer detection using exhaled breath analysis [3,4,5,6]. The median HCOH level observed for lung cancer patients (83 ppb) is higher than that for healthy ones (48 ppb) [5]. Thus, both medical and air quality applications require the detection of formaldehyde in the sub-ppm range.

Metal oxide based semiconductor gas sensor for formaldehyde detection are quite attractive since they can have sufficient sensitivity, are inexpensive and can be integrated into portable devices such as e-Nose, that could detect formaldehyde at ppb level at high humidity level (e.g., 90% RH for breath analysis) and in the presence of different interfering gases [6,7,8]. However, most studies consider the sensor characteristics of various semiconductor oxides in the detection of HCHO in the concentration range of 10 ppm or more [9,10]. Recent efforts are focused at the implementation of a new principle of HCHO detection under conditions of minimal thermal heating combined with illumination by a low-power UV or visible light source [11,12].

It was shown by the authors of [13,14,15,16,17,18] that under UV illumination molecules of volatile organic compounds (VOCs: acetone, acetaldehyde, ethanol, hydrocarbons) undergo photolysis on the surface of semiconductor oxides that facilitates their subsequent oxidation with chemisorbed oxygen, leading to a change in the conductivity of the semiconductor. Titanium dioxide TiO_2_ and zinc oxide ZnO demonstrate the highest activity in the photocatalytic VOCs oxidation. A comparative study on UV light activated porous TiO_2_ and ZnO sensors demonstrated that TiO_2_ exhibited a superior performance to ethanol and formaldehyde [19].

As a sensor material for HCHO detection TiO_2_ with different morphology was studied: mesoporous powders [20], nanotube arrays [21], polycrystalline thick films [22], hollow microspheres [23]. In all cases, the investigations were conducted under UV illumination. The experimental study of the TiO_2_ sensor properties in dark conditions is difficult because of the exceptionally high electrical resistance of these materials. A decrease in the resistance of TiO_2_-based materials can be achieved by creating nanocomposites, in which TiO_2_ plays the role of a photocatalyst/receptor, while the transport of charge carriers is effectuated by another semiconductor with more suitable electrical characteristics. Nanocomposites SnO_2_/TiO_2_ are promising candidates for formaldehyde detection both in dark conditions and under UV illumination. It was shown that as compared with pure SnO_2_ and TiO_2_, nanocomposites with different Sn/Ti ratio demonstrate higher sensor response toward triethylamine [24], ammonia [25], hydrogen [26,27], and formaldehyde [28]. Moreover, DFT calculations and experimental study presented by the authors of [29] revealed that these nanocomposites have higher photocatalytic activity in UV-light initiated degradation of RB5 and RhB dyes. However, the role of each component in the formation of the sensor signal when detecting formaldehyde in dark conditions and under UV illumination is not clear.

In this paper, we compare the sensor properties of nanocrystalline SnO_2_ and TiO_2_@SnO_2_ nanocomposites when detecting formaldehyde in the sub-ppm range in dark conditions and under periodic UV illumination in the temperature range of 50–300 °C.

## 2. Materials and Methods

### 2.1. Materials Synthesis

Nanocrystalline SnO_2_ was prepared by the precipitation method. Aqueous ammonia solution (25%) was added dropwise to 0.3 M solution of tin (IV) chloride pentahydrate (SnCl_4_·5H_2_O, 98%, Sigma-Aldrich) under vigorous stirring until pH = 7 [30,31]. The synthesis was carried out at room temperature. The obtained α-stannic acid gel was separated by centrifugation at 3500 rpm for 3 minutes, repeatedly washed with deionized water and then with 0.01 M NH_4_NO_3_ solution. AgNO_3_ test was used to confirm the negative reaction to chloride ions. The resulting gel was dried in air at 50 °C for 24 h, then obtained β-stannic acid xerogel was ground in an agate mortar and annealed in air at 300 °C for 24 h. According to transmission electron microscopy data [30,31] the SnO_2_ powder consists of a rounded shape, agglomerated nanoparticles of 3–8 nm in diameter.

Nanocomposites TiO_2_@SnO_2_ were synthesized by atomic layer deposition (ALD) method. Titanium (IV) isopropoxide (Ti(OCH(CH_3_)_2_)_4_, TTIP) and H_2_O used as precursors, were introduced consequently into the growth reactor containing SnO_2_ nanocrystals to avoid premature interaction. The ALD parameters: temperature and pressure were set to 150 °C and 0.1 torr, respectively. TTIP and H_2_O were kept in bubblers at 40 °C and 24 °C, respectively. The typical ALD cycle included the following stages: 1 s for TTIP dosing, 10 s for N_2_ purging, 0.2 s for H_2_O dosing, and 25 s for N_2_ purging. The ALD cycles were repeated for 500, 1000 and 2000 times, respectively, producing TiO_2_@SnO_2_ nanocomposites with increasing Ti/Sn ratio. After ALD synthesis the nanocomposites were annealed in air at 500 °C for 1 h to remove organic residues and ensure TiO_2_ crystallization. To obtain an adequate reference sample, nanocrystalline SnO_2_, which was not subjected to ALD treatment, was additionally annealed in air at 500 °C for 24 h.

### 2.2. Materials Characterization

Chemical composition ([Ti]/([Ti]+[Sn]), mol%) of TiO_2_@SnO_2_ nanocomposites was determined by X-ray fluorescent analysis (XRF) performed on M1 Mistral spectrometer (Bruker, Billerica, MA, USA) with the beam energy of 50 keV. The diameter of the analyzed area was 1.0 mm, the signal accumulation time 2 min.

The microstructure of the synthesized products was studied by high resolution transmission electron microscopy (FE-TEM, JEOL JEM-2100F, JEOL Ltd., Tokyo, Japan).

The phase composition of TiO_2_@SnO_2_ nanocomposites was characterized using powder X-ray diffraction (XRD) and Raman spectroscopy. The XRD patterns were collected by DRON-4 diffractometer (Burevestnik, Moscow, Russia) using monochromatic Cu Kα radiation (λ = 1.5406 Å). The survey was carried out in the range of 2θ = 10°–80° with a step of 0.1° at scanning rate of 0.5°/min. The crystallite size *d_XRD_* of SnO_2_ and Ti_x_O_y_ phases was calculated from the broadening of the most intense XRD peaks using Scherer equation. WinXPow (STOE and Cie GmbH, Darmstadt, Germany) software was used for phase and full profile analysis, DSH software (laboratory made) was used for crystallite size calculations. The crystalline phases were determined using structural parameters of SnO_2_ cassiterite (ICDD 41-1445), TiO_2_ anatase (ICDD 21-1272) and TiO_2_ brookite (ICDD 75-1582). Raman spectra were recorded by a Renishaw InVia multichannel spectrometer (Renishaw plc., Gloucestershire, UK) using an argon laser with a wavelength of 514 nm as the radiation source. The laser beam was focused on the sample using a microscope objective (magnification × 50) so that the analyzed area was approximately 100 μm^2^. The spectra were recorded in air at room temperature in the range of 100–900 cm^−1^ with a step of 1 cm^−1^ (data was collected for each point for 10 s).

The nature of chemical groups presenting in obtained samples was investigated using the FT-IR spectroscopy. The IR spectra were recorded using a Perkin–Elmer Spectrum One Fourier Transform Infrared (FT-IR) spectrometer (Perkin Elmer Inc., Waltham, MA, USA) in the range of 4000–400 cm^–1^ with a step of 1 cm^–1^ in the transmission mode. For this the TiO_2_@SnO_2_ powders (0.3–0.5 mg) were grinded with 50 mg of dried (350 °C, 2 h) KBr (FT-IR grade, Sigma-Aldrich, Saint Louis, MO, USA) and pressed into tablets. DRIFT spectra were collected in situ using the DiffusIR annex and heated flow chamber HC900 (Pike Technologies, Fitchburg, WI, USA) sealed by ZnSe windows. DRIFT spectra were registered in the range 4000–1000 cm^−1^ at 4 cm^−1^ resolution with an accumulation of 30 scans. Powders (50 mg) were placed in alumina crucibles (5 mm diameter). Gas mixture containing 100 ppm of HCHO in dry air was used for the investigations.

The investigations of SnO_2_ reference sample and TiO_2_@SnO_2_ nanocomposites by the method of thermo-programmed reduction with hydrogen (TPR-H_2_) was effectuated on Chemisorb 2750 (Micromeritics, Norcross, GA, USA) in a quartz reactor using 10% H_2_/Ar gas mixture (50 mL/min) under the heating to 900 °C at a heating rate of 10 K/min.

**Figure 1:**
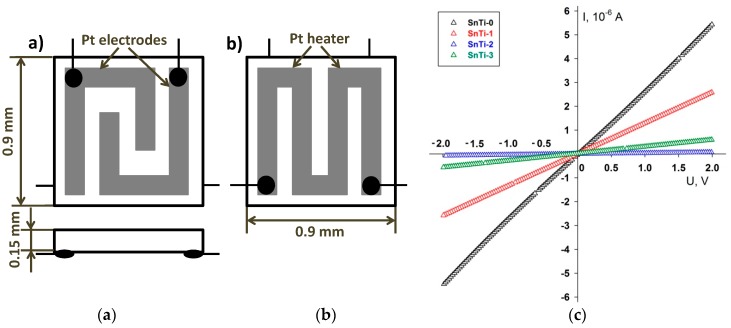
Micro-hotplate scheme: (**a**) front side; (**b**) back side; (**c**) Current–voltage (I-V) characteristics of the sensors.

Specially designed micro-hotplates were used to investigate gas sensor properties. The micro-hotplates consist of dielectric substrate (Al_2_O_3_) with dimensions of 0.9 × 0.9 × 0.15 mm, which provides a small temperature gradient between the heater and the sensitive layer. Substrate is covered with Pt electrodes on the top side for resistance measurements and Pt heater on the back side, which is protected by an insulating layer of dielectric paste. Electrodes and heater are made using Pt-based paste by screen printing method (Figure 1). The powders of nanocrystalline oxides were mixed with a vehicle (α-terpineol in ethanol) and deposited in the form of thick films over dielectric substrate to cover the electrodes. Thick films were sintered at 300 °C for 10 hours in air to remove the organic binder. The method used allows us to obtain the coatings which are continuous and uniform over the entire substrate with the thickness about 1 μm [30] and with the value of resistance in air at 300 °C, differing by no more than 10%. The current–voltage (I-V) characteristics of the sensors measured using Potentiostat P-8-NANO (Elins, Zelenograd, Russia) are shown in Figure 1c. All the samples exhibited linear I-V curves both for positive and negative applied bias voltages up to +2V and −2V, respectively.

**Figure 2:**
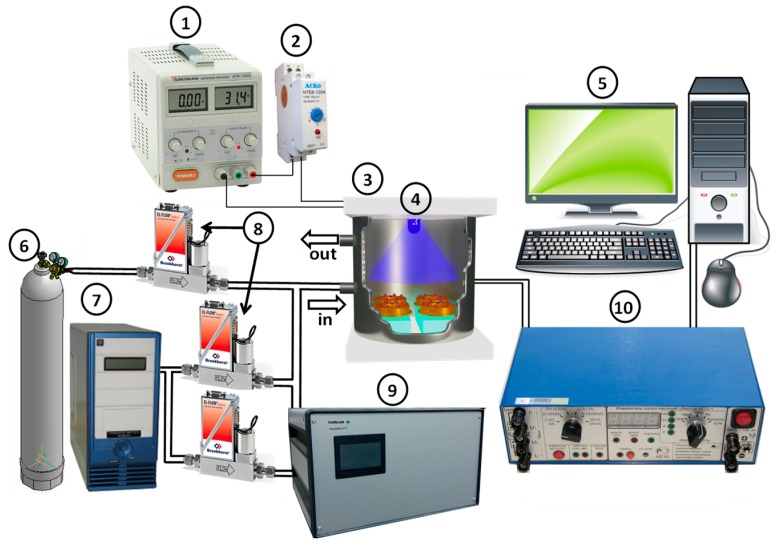
Schematic illustration of sensor measurements setup. (1) DC power supply QJ2002C (Ningbo JiuYuan Electronic, Zhejiang, China); (2) Time relay REV-114 (Novatek-Electro, Moscow, Russia); (3) Teflon airtight and light-tight chamber (laboratory-made); (4) UV LED (SMD-5050 XML, Epileds Co. LTD, Taiwan, China); (5) Control PC; (6) Analyte gas bottle (4.6 ppm of HCOH in N_2_, certified gas mixture, Monitoring, Russia); (7) Pure air generator («Granat-Engineering» Co.Ltd, Moscow, Russia); (8) Mass Flow Controller EL-FLOW F-221M (Bronkhorst, Ruurlo, The Netherlands); (9) Humidifier Cellkraft P-2 (Cellkraft AB, Stockholm, Sweden); (10) electronic module providing control of sensor heating and high-precision measurement of the resistance of the sensitive layer (MGA-2-1, Senseria, Tomsk, Russia).

The schematic illustration of sensor measurements setup is shown in Figure 2. The DC conductivity was measured in situ using electronic module (10) providing control of sensor heating and high-precision measurement of the resistance of the sensitive layer. The sensors were placed into a Teflon airtight and light-tight flow chamber (3) connected to a computer-controlled (5) gas delivery system with electronic mass-flow controllers (8). The measurements were carried out under a controlled constant flux of 100 ml/min in the temperature range of 300–50 °C with a step of 50 °C, in the dark conditions and under periodic UV illumination. Miniature UV LED (4) (10 mW/cm^2^, λ_max_ = 365 nm) located at a distance of 4 cm above the sensors was used as an illumination source. The power of the LED was effectuated using DC power supply (1). The illumination of the sensors was carried out in a pulsed mode with the period 2 min “on”–2 min “off”. An automatic cyclic relay (2) periodically opened and closed the circuit, allowing measurements to be made with periodic illumination. The sensor properties of SnO_2_ reference sample and TiO_2_@SnO_2_ nanocomposites were investigated toward formaldehyde HCHO (0.06–0.08–0.15–0.3–0.6 ppm) in air (relative humidity at 25 °C RH_25_ = 30%). The gas mixtures containing preassigned concentration of HCHO were prepared by dilution of certified gas mixture (6) with background purified air (7). Flow through humidifier (9) was used to adjust the humidity (RH_25_ = 30%) of gases passing through the sensor chamber. The sensor response was calculated as $S=R_{air}/R_{gas}$, where $R_{air}$ is the resistance in background air, $R_{gas}$ is the resistance in the presence of HCHO.

## 3. Results and Discussion

### 3.1. Characteristics of Nanocrystalline SnO_2_ and TiO_2_@SnO_2_ Nanocomposites

The elemental and phase composition of the samples under investigation are presented in Table 1. An increase in the number of ALD cycles led to a proportional increase in the titanium content (presented as ([Ti]/([Ti]+[Sn]) ratio, mol%) in nanocomposites.

**Figure 3:**
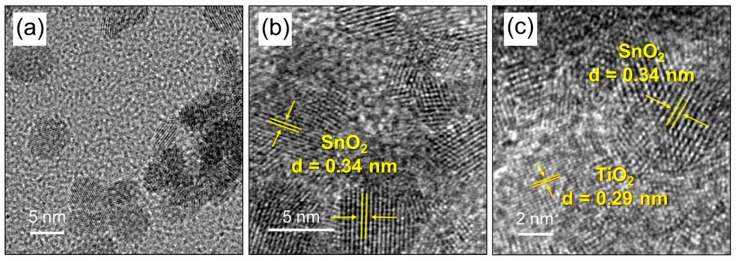
(**a**) TEM and (**b**) Lattice resolved TEM image of SnO_2_ powder used as a substrate in ALD synthesis; (**c**) Lattice-resolved TEM image of SnTi-1 nanocomposite.

Figure 3 shows the TEM image of SnO_2_ powder used as a substrate in ALD synthesis confirming formation of SnO_2_ nanocrystals with approximate size of 5–10 nm. Lattice-resolved TEM image presented in Figure 3b highlights the crystalline phase of SnO_2_ with a plane spacing of 0.34 nm belonging to the (110) plane of cassiterite SnO_2_. The lattice-resolved TEM image of SnTi-1 nanocomposite (Figure 3c) makes it possible to detect crystalline regions with interplanar distances of 0.34 and 0.29 nm, corresponding to (110) SnO_2_ (cassiterite) and (121) TiO_2_ (brookite), respectively.

**Figure 4:**
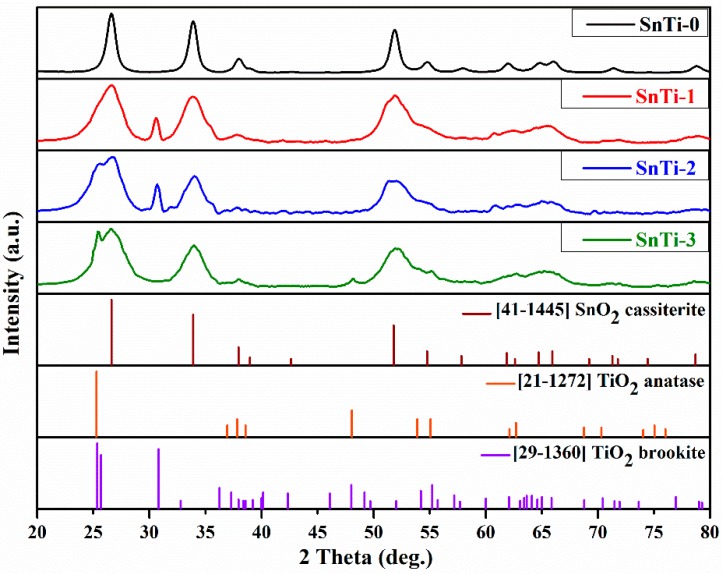
XRD patterns of nanocrystalline SnO_2_ and TiO_2_@SnO_2_ composites and references for SnO_2_ cassiterite (ICDD 41-1445), TiO_2_ anatase (ICDD 21-1272) and TiO_2_ brookite (ICDD 29-1360).

According to the results of the XRD analysis (Figure 4), the SnO_2_ annealed at 500 °C (equivalently to the post-synthetic annealing used for the synthesis of TiO_2_@SnO_2_ nanocomposites) crystallizes in a tetragonal cassiterite structure (ICDD 41-1445) with a crystallite size of 9–10 nm. Intense reflections from the cassiterite phase are observed in the diffraction patterns of all the samples. The titanium-containing phases, formed during post-synthetic annealing, change with an increase in the number of ALD cycles. The TiO_2_ with brookite structure (ICDD 29-1360) presents in SnTi-1 and SnTi-2 nanocomposites, as evidenced by the appearance of the intense (121) diffraction peak at 2θ = 30.8°. The most intense (120) at 2θ = 25.3° and (111) 2θ = 25.7° diffraction reflections of brookite coincide with the most intense (101) diffraction reflection (2θ = 25.3°) of the TiO_2_ anatase phase (ICDD 21-1272). Therefore, the presence of the anatase phase in these nanocomposites cannot be excluded. The intensity of diffraction peak at 2θ = 25.3° increases with increasing titanium content. In the diffraction pattern of SnTi-3 nanocomposite a new diffraction peak at 2θ = 48.1° corresponds to the (200) reflection of the anatase phase. At the same time, the diffraction peak at 2θ = 30.8° does not appear that indicates the absence of the brookite phase in this sample. The least intense and wide peaks in the ranges of 2θ = 36.5°–40.0° and 2θ = 52.6°–64.5° correspond to the superposition of the reflections of almost all of the above phases, so their deconvolution and assignment to a certain phase is a difficult task. The broadening of the SnO_2_ reflections in the diffraction patterns of nanocomposites and overlap of intense diffraction peaks corresponding to SnO_2_ and TiO_2_ in SnO_2_-TiO_2_ (anatase) and SnO_2_-TiO_2_ (brookite) systems (in contrast to the TiO_2_-ZnO [32]) do not allow us to make a positive or negative conclusion about the incorporation of Ti into the SnO_2_ lattice.

The crystallite size of detected Sn- and Ti-containing phases depending on titanium content in the samples is presented in Table 1. The crystallite size of tin dioxide, used as a substrate for the deposition of titanium dioxide by the ALD method, is 3–4 nm [30,31]. The post-synthetic annealing at 500 °C, necessary for the removal of organic residues of the titanium precursor, leads to an increase in the size of SnO_2_ crystallites to 9–10 nm. With an increase in the titanium content in TiO_2_@SnO_2_ nanocomposites, a decrease in the size of SnO_2_ crystal grains is observed as compared with the reference sample. A similar effect was repeatedly noted for nanocomposites based on semiconductor metal oxides [33,34,35]. The presence of impurities on the surface of growing crystallites slows down their growth rate under isothermal annealing due to the so-called Zener pinning [36]. The maximum crystallite size of the main phase is determined by the volume fraction and the size of particles (crystalline or amorphous) segregated on the surface of growing crystallites. The deposition of TiO_2_ layer on the surface of nanocrystalline SnO_2_ reduces the area of SnO_2_ intergranular contacts, that prevents recrystallization of SnO_2_ particles. The greater the thickness of the deposited TiO_2_ layer, the less the coarsening of SnO_2_ particles occurs. For the TiO_2_ brookite phase in SnTi-1 and SnTi-2 nanocomposites the crystallite size was estimated using the (121) reflection that does not overlap with another diffraction peaks. For the size of the crystallites of the TiO_2_ anatase phase, such an estimate is possible only in the case of SnTi-3 nanocomposite, in which there is no brookite phase. For the Ti-containing phases, the increase in the crystallite size is observed. Since during the ALD cycles the Ti-containing precursor has been deposited on the outer surface of SnO_2_ agglomerates, the sintering and coarsening of Ti-containing particles will be easier. That is why an increase in the titanium content leads to an increase in the crystallite size of the Ti-containing phases.

**Figure 5:**
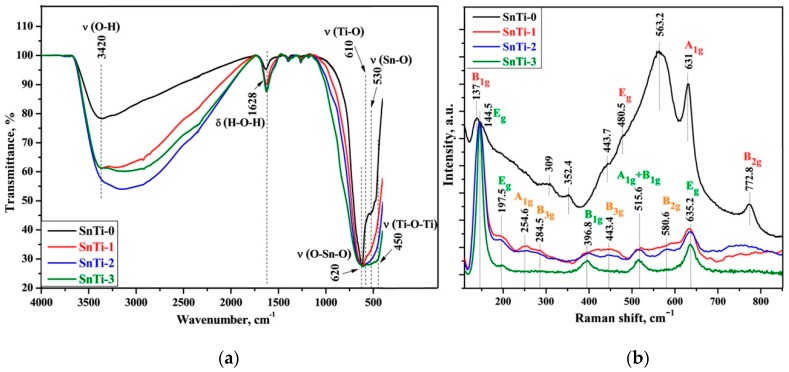
(**a**) FT-IR spectra of nanocrystalline SnO_2_ and TiO_2_@SnO_2_ composites normalized to the intensity of (O–Sn–O) oscillations; (**b**) Normalized Raman spectra of nanocrystalline SnO_2_ and TiO_2_@SnO_2_ composites.

The IR spectra of nanocrystalline SnO_2_ and TiO_2_@SnO_2_ composites are compared in Figure 5a. In all spectra, a large broad peak in the region of 2700–3500 cm^–1^ is observed, which is related to the stretching vibrations of OH groups and a peak at 1628 cm^–1^, which is related to the deformation vibrations of adsorbed water [37,38]. The wide and intense absorption bands at 530 cm^–1^ and 620 cm^–1^ in the case of SnO_2_ correspond to stretching vibrations of the Sn–O and symmetric vibrations of the O–Sn–O bonds, respectively [39]. The metal–oxygen (M–O) oscillation modes for SnO_2_ and TiO_2_ in TiO_2_@SnO_2_ composites overlap in the range of 400–650 cm^–1^, nevertheless different components can be distinguished. All the FT-IR spectra of the composites show vibration modes at 450 and 610 cm^–1^, which can be attributed to oscillations of Ti–O–Ti and Ti–O bonds, respectively [40,41,42]. With an increase in the titanium content an increase in the intensity of the Ti–O oscillation modes is observed, which appears as a broadening of the M–O absorption peak in this region.

Figure 5b shows the Raman spectra of nanocrystalline SnO_2_ (SnTi-0) and TiO_2_@SnO_2_ composites. Raman spectrum of the SnO_2_ clearly shows three characteristic modes at 480.5, 631 and 772.8 cm^−1^ that correspond to the E_g_, A_1g_ and B_2g_ vibrational modes, respectively [43,44]. The A_1g_ and B_2g_ modes are associated with symmetric and asymmetric Sn–O stretching, respectively, orthogonally to the *c* axis. The translational E_g_ mode is related to the motion of oxygen anions along the *c* axis [43,44]. The B_1g_ oscillation mode appears only in the spectra of nanocrystalline SnO_2_ in the range of 100–184 cm^−1^ [45,46]. In the SnO_2_ spectrum presented in Figure 5b the band at 137 cm^−1^ is due to B_1g_ mode, the shift may be associated with the nanoparticles size effect.

J. Zuo et al. studied the size effects in SnO_2_ nanoparticles [47]. They showed that, in addition to the characteristic vibrational modes of bulk SnO_2_, the Raman spectrum of nanocrystalline SnO_2_ has two additional Raman scattering bands at 358 (B_1_) and 572 cm^−1^ (B_2_). The B_2_ band corresponds to surface modes and is very sensitive to the changes in crystallite size for nanoscale SnO_2_. The appearance of surface modes is associated with a small particle size of SnO_2_ and can be due to the appearance of oscillations that are forbidden by symmetry due to the breaking of long-range order in the systems of reduced dimension. That is why a decrease in crystallite size leads to the formation of a highly defective surface layer, the contribution of which will be the highest for the materials with the smallest particle size [48]. Based on the above considerations, the wide band located at 563 cm^−1^ corresponds to the surface modes associated with in-plane oxygen vacancies of the nanocrystalline cassiterite SnO_2_ [48,49,50]. Consequently, the small size and surface defects of the SnO_2_ nanoparticles may have a positive effect on the gas sensitivity of the sensor. This assumption was experimentally confirmed in by the authors of [31], where it was shown that the relative intensity of Raman surface modes *I_S_/I_V_* taken as the ratio of the sum of their intensities *I_S_* to the intensity of A_1g_ mode *I_V_* demonstrates the best linear correlation with gas response of SnO_2_ nanocrystalline materials to CO.

Changes in the crystal’s local symmetry produce changes in some of the components of the polarizability tensor, even for usually forbidden vibration modes [49]. That is why the A_2u_ IR active and Raman forbidden modes are found to transform into Raman active modes [48]. In this case the bands at 309 and 352 cm^−1^ (E_u_) and the band at 444 (B_1u_) are related to transformation of an IR to Raman active modes.

Raman peaks observed in the spectra of TiO_2_@SnO_2_ composites at 145, 198, 397, 516 and 635 cm^−1^ (Figure 5b), refer to the E_g_, E_g_, B_1g_, A_1g_ + B_1g_ and E_g_ modes of the anatase phase, respectively [51]. For TiO_2_ nanoparticles the E_g_ Raman peak is mainly caused by symmetric stretching vibration of O–Ti–O groups, B_1g_ peak is caused by symmetric bending vibration of O–Ti–O and A_1g_ peak is caused by antisymmetric bending vibration of O–Ti–O [52]. The presence of an intense 145 cm^−1^ mode (a characteristic oscillation mode of anatase) indicates that TiO_2_ nanocrystals have a certain degree of long-range order, while weaker and wider peaks in the high-frequency region indicate the absence of a short-range order in the anatase phase [53,54,55]. According to the factor group analysis, TiO_2_ brookite phase has a total of 36 Raman active modes (9A_1g_ + 9B_1g_ + 9B_2g_ + 9B_3g_). Raman spectra of the SnTi-1 and SnTi-2 composites show both anatase and brookite bands. In total, 4 brookite bands were readily identified, including A_1g_ (255 cm^−1^), B_3g_ (285, 443 cm^−1^), B_2g_ (581 cm^−1^). Also, the A_1g_ (153, 194 cm^−1^) and B_2g_ (395 cm^−1^) brookite bands may be overlapped by anatase modes, which are very close to them [56,57]. The full assignment of the vibrational modes in the Raman spectra of nanocomposites is presented in Table 2.

Thus, the Raman spectra confirm the data obtained by the XRD method. The formation of the metastable brookite phase is observed in SnTi-1 and SnTi-2 composites. Anatase can play the role of a stabilizer for this phase [58]. A correlation among the surface enthalpies of the TiO_2_ three polymorphs and their particle size was found by Zhang and Banfield [59]. The formation energies of anatase, brookite and rutile are sufficiently close that they can be reversed by small differences in surface energies. Zhu et al. [60] developed an empirical expression on a critical grain size of brookite, which determines the transition sequence between anatase and brookite. These transformations become noticeable with prolonged isothermal annealing at temperatures above 500 °C. To avoid the changes in phase composition and crystallite size of nanocomposites subjected to post synthesis annealing at 500 °C, the maximum temperature during the manufacture of sensor elements and gas sensor measurements did not exceed 300 °C.

### 3.2. Gas Sensor Properties

As discussed in the review [8], depending on the operating temperature the change of the resistance of *n*-type semiconductor oxides when interacting with formaldehyde is due to HCHO oxidation with chemisorbed oxygen $O_{\beta \left(ads\right)}^{\alpha -}$ to HCOOH or CO_2_:

(1) $$\beta \cdot HCHO_{\left(gas\right)}+O_{\beta \left(ads\right)}^{\alpha -}=\beta \cdot HCOOH_{\left(gas\right)}+\alpha \cdot e^{-}$$

(2) $$\beta \cdot HCHO_{\left(gas\right)}+2O_{\beta \left(ads\right)}^{\alpha -}=\beta \cdot CO_{2\left(gas\right)}+\beta \cdot H_{2}O_{\left(gas\right)}+2\alpha \cdot e^{-}$$

At constant temperature and HCHO concentration, the value of the sensor response will depend on the concentration and the predominant form of chemisorbed oxygen on the surface of the semiconductor oxide.

According to the literature [20,61,62,63,64], the interaction of UV light with TiO_2_ involves the following processes: electron-hole pairs generation (3), oxygen photodesorption (4), formation of “active” chemisorbed oxygen (5), which is able to oxidize formaldehyde even at room temperature (6)

(3) $$h\nu \overset{TiO_{2}}{\to }h^{+}\left(h\nu \right)+e^{-}\left(h\nu \right)$$

(4) $$h^{+}\left(h\nu \right)+O_{2\left(ads\right)}^{-}\to O_{2\left(gas\right)}$$

(5) $$O_{2\left(gas\right)}+e^{-}\left(h\nu \right)\to O_{2\left(ads\right)}^{-}\left(h\nu \right)$$

(6) $$HCHO_{\left(gas\right)}+O_{2\left(ads\right)}^{-}\left(h\nu \right)\to CO_{2\left(gas\right)}+H_{2}O_{\left(gas\right)}+e^{-}$$

Under UV light, hydroxyl groups presented on the TiO_2_ surface can also pass into the “active” form (7) and then participate in the oxidation of formaldehyde (8):

(7) $$OH^{-}+h^{+}\left(h\nu \right)\to OH^{•}\left(h\nu \right)$$

(8) $$HCHO_{\left(gas\right)}+2\cdot OH^{•}\left(h\nu \right)\to CO_{2\left(gas\right)}+H_{2}O_{\left(gas\right)}+2\cdot H^{+}+2\cdot e^{-}$$

**Figure 6:**
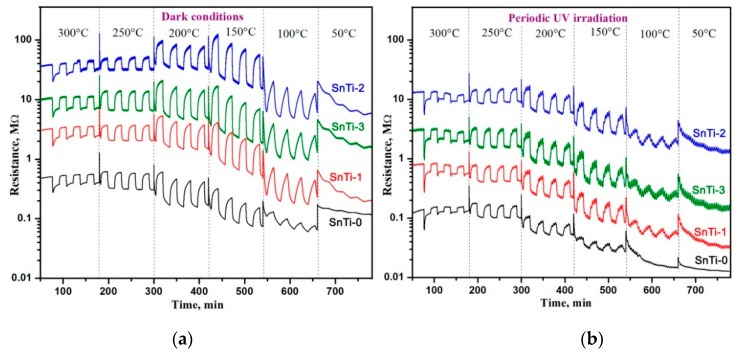
Resistance of the TiO_2_@SnO_2_ nanocomposites in the temperature range 300–50 °C under periodic change of the gas phase composition (**a**) in dark conditions; (**b**) under periodic UV (λ_max_ = 365 nm) illumination.

Figure 6 demonstrates the change in the resistance of nanocomposites with the cyclic changes in the composition of the gas phase “air (15 min)”–“0.6 ppm HCHO in air (15 min)”. The measurements were carried out in the temperature range of 300–50 °C in dark conditions (Figure 6a) and under periodic UV illumination (Figure 6b). At a fixed temperature, TiO_2_@SnO_2_ nanocomposites have a higher resistance than unmodified SnO_2_ (SnTi-0). UV illumination leads to reduced resistance of nanocomposites by 1.5–3 times. In all cases in the presence of HCHO, the resistance of SnO_2_ and TiO_2_@SnO_2_ nanocomposites decreases due to the oxidation of formaldehyde by chemisorbed oxygen. In dark conditions at low measurement temperatures (T < 200 °C), the baseline resistance drift is observed for all samples. This may be due to the accumulation of formaldehyde oxidation products on the surface of the sensitive layer under these conditions. The use of UV illumination reduces the drift of the resistance at T = 150 °C. It can be assumed that UV light stimulates the desorption of the products of formaldehyde oxidation.

**Figure 7:**
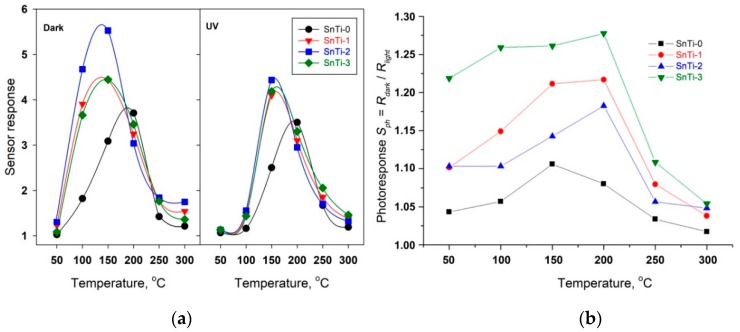
(**a**) Temperature dependencies of sensor response of the TiO_2_@SnO_2_ nanocomposites toward 0.6 ppm HCHO in air in dark conditions (left) and under UV periodic illumination (right); (**b**) Temperature dependencies of effective photoresponse *S_Ph_* of the TiO_2_@SnO_2_ nanocomposites in background air.

The obtained results allowed us to draw the temperature dependence of the sensor response $S=R_{air}/R_{gas}$ (Figure 7a). The maximum sensor response of unmodified SnO_2_ (SnTi-0) is observed at T = 20 °C. The introduction of Ti-containing phases leads to a decrease in the temperature of the maximum sensor signal to 150 °C. In dark conditions, the maximum response was detected for the SnTi-2 nanocomposite. The use of UV illumination does not change the position of the maxima on the temperature dependence of the sensor response, but unexpectedly it leads to a slight decrease in the signal value in the low temperature range of 50–150 °C. Such a decrease in the sensor response can be caused by a decrease in the concentration of chemisorbed oxygen participating in the oxidation of formaldehyde by reactions (1) and (2), due to the partial desorption of oxygen from the surface of semiconductor oxides under UV light [65]. Figure 7b shows the temperature dependences of the effective photoresponse *S_Ph_* of nanocomposites in background air calculated as *S_Ph_* = *R_dark_*/*R_light_*, where *R_light_* is the minimum resistance achieved during the sensor illumination, and *R_dark_* is the maximum resistance achieved in the dark period [66]. The maximum photoresponse corresponds to a temperature range of 150–200 °C. A decrease in the *S_Ph_* value with an increase in the measurement temperature up to 250–300 °C is due to the contribution of thermal oxygen desorption in dark conditions.

**Figure 8:**
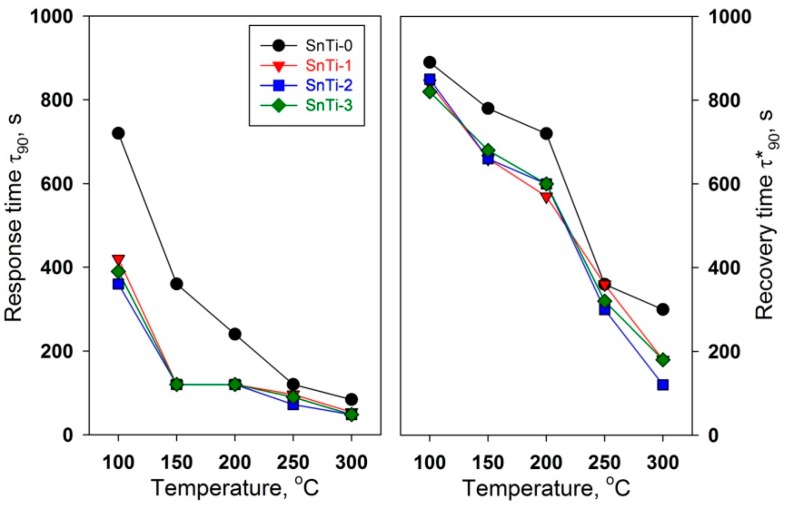
Temperature dependencies of response time $\tau_{90}$ (left) and recovery time $\tau_{90}^{*}$ (right) during detection of 0.6 ppm HCHO in air in dark conditions.

The dynamic sensor characteristics, response time $\tau_{90}$. and recovery time $\tau_{90}^{*}$. , are presented in Figure 8. Even though the absolute values of response time and recovery time are strongly dependent on the parameters of the testing system (geometry and size of the measurement cell, the method to switch the gases, the gas flow rate), they are useful to compare the dynamic sensor characteristics of materials if measurements are performed in identical conditions. With a decrease in the operating temperature, an increase in both the response and recovery times is observed. It should be noted that the sensor based on unmodified SnO_2_ is characterized by significantly worse dynamic characteristics than sensors based on TiO_2_@SnO_2_ nanocomposites, which are close to each other. Such a difference may be due to a decrease in the degree of sintering of SnO_2_ nanoparticles in nanocomposites as compared to unmodified tin dioxide.

**Figure 9:**
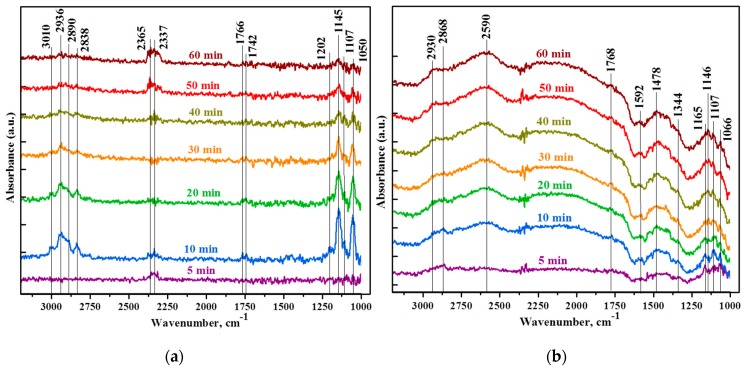
In situ DRIFT spectra of the unmodified SnO_2_ (SnTi-0) (**a**) and SnTi-2 nanocomposite (**b**) during HCHO adsorption at room temperature.

To clarify the mechanism of formaldehyde oxidation on the surface of semiconductor oxides, the in situ DRIFT studies have been conducted. In situ DRIFT spectra of the unmodified SnO_2_ (SnTi-0) and SnTi-2nanocomposite during HCHO adsorption at room temperature are shown in Figure 9.

The bands of weakly bonded forms at 1202, 1742, 1766 and 3010 cm^−1^ for SnTi-0 and at 1768 cm^−1^ for SnTi-2 attributed to molecular adsorption form of HCHO with the formation of hydrogen bonds between its carbonyl oxygen and surface hydroxyl groups [67,68,69]. The formation of CO_2_ (at 2337 and 2365 cm^−1^) was observed on SnTi-0 after 5 min of HCHO adsorption, moreover these bands grew in intensity with the increase of the adsorption time [70]. In SnTi-2 spectra the bands at 1344 and 1592 cm^−1^ are assigned to COO symmetric stretching and COO asymmetric stretching of formate species [71,72]. The bands located at 2838, 2890 and 2590, 2868 cm^−1^ for SnTi-0 and SnTi-2, respectively, belong to CH stretching of formate species [68,73,74]. The bands at 2936 cm^−1^ for SnTi-0 and 2930 cm^−1^ for SnTi-2 were assigned to the characteristic peak of dioxymethylene (DOM) intermediate [71]. The other DOM bands also were identified at 1050, 1107, 1145 cm^−1^ for SnTi-0 and 1066, 1107, 1146, 1165, 1478 cm^−1^ for SnTi-2 [67,68].

**Figure 10:**
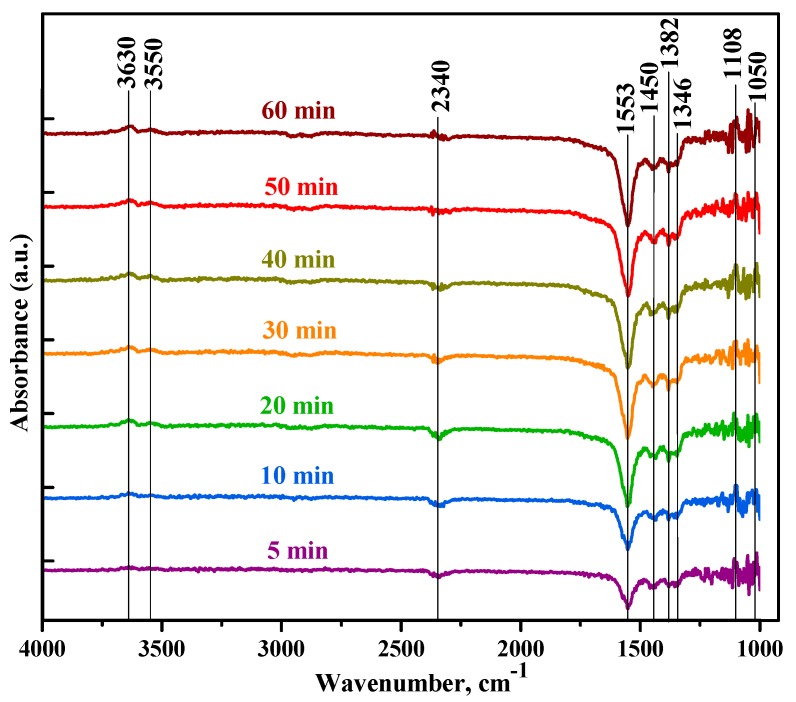
In situ DRIFT spectra of the SnTi-2 nanocomposite obtained during the heating in dry air at T = 300 °C after formaldehyde adsorption at room temperature.

Figure 10 shows the DRIFT spectra of the SnTi-2 nanocomposite obtained during the heating in dry air at T = 300 °C after formaldehyde adsorption at room temperature. From the results, it can be seen that the formats are intensively desorbed from the surface of the nanocomposite, as evidenced by decreasing of their characteristic vibrational modes at 1346, 1382 and 1553 cm^−1^ [68,71,72]. However, at such a high temperature, there is an increase in the intensity of dioxymethylene species, the bands of which are at 1050, 1108 and 1450 cm^−1^ [67,68], which indicates partially HCHO oxidation to DOM. Most likely, at T=300 °C, formaldehyde is oxidized to CO_2_ and H_2_O, as a result of which hydroxyl groups of water accumulate on the surface (increase in the intensity of OH bands at 3550 and 3630 cm^−1^) and CO_2_ desorption occurs (decrease in intensity at 2340 cm^−1^); at room temperature CO_2_ is accumulated on the SnTi-0 surface (Figure 9a). The assignment of IR bands for intermediates is summarized in Table 3.

So, for HCHO oxidation, oxygen ions adsorbed on the surface of the nanocomposites play a key role in the generation of the surface formates. According to the results of the in situ DRIFT analysis and literature review [67,68,69,70,75,76] we propose a reaction mechanism as depicted in (9)–(12):

(9) $$HCHO_{\left(gas\right)}\to HCHO_{\left(ads\right)}$$

(10) $$\beta \cdot HCHO_{\left(ads\right)}+O_{\beta \left(ads\right)}^{\alpha -}\to \beta \cdot H_{2}COO_{\left(ads\right)}^{-}+\left(\alpha -1\right)\cdot e^{-}$$

(11) $$\beta \cdot H_{2}COO_{\left(ads\right)}^{-}+O_{\beta \left(ads\right)}^{\alpha -}\to \beta \cdot HCOO_{\left(ads\right)}^{2-}+\beta \cdot OH_{\left(ads\right)}^{\left(\alpha -1\right)-}$$

(12) $$2\beta \cdot HCOO_{\left(ads\right)}^{2-}+O_{\beta \left(ads\right)}^{\alpha -}\to 2\beta \cdot CO_{2}+\beta \cdot H_{2}O+\left(4\beta +\alpha \right)\cdot e^{-}$$

At first, formaldehyde molecularly adsorbs as a whole via strong hydrogen-bonding interactions (9). Desorption of formaldehyde is in competition with the reaction between formaldehyde and co-adsorbed surface oxygen to yield formate HCOO-, which can proceed via formation of dioxymethylene H_2_COO^-^ intermediate (10),(11) [69]. For SnTi-0 sample there are two weak bands at 2337 and 2365 cm^−1^ indicated the appearance of carbon dioxide [64,77]. In this case, CO_2_ and H_2_O were formed as the result of oxidation of formate ions (12) of chemisorbed oxygen on semiconductor oxides [78], reaction (10) proceeds without changing the electron concentration in the conduction band of the semiconductor, that causes the absence of a sensor response at T = 50 °C (Figure 6).

**Figure 11:**
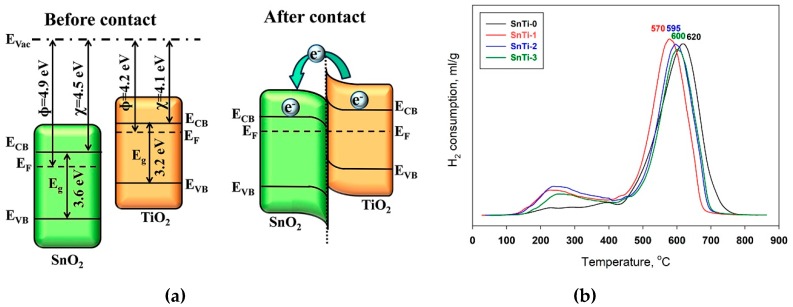
(**a**) Estimated band alignment of the SnO_2_ (cassiterite) and TiO_2_ (anatase) phases (left) and SnO_2_/TiO_2_ *n*-*n* heterocontact (right); (**b**) TPR-H_2_ profiles of unmodified SnO_2_ (SnTi-0) and TiO_2_@SnO_2_ nanocomposites.

The observed effect of Ti-containing phases on the sensor response of SnO_2_ toward HCHO in dark conditions should be explained based on the model considering the formation of *n*-*n* heterocontact at the SnO_2_/TiO_2_ interface. The estimated band alignment of the SnO_2_ (cassiterite) and TiO_2_ (anatase) is presented on Figure 11a [24,79,80]. The available lower-energy conduction band states stimulate electron transfer to *n*-SnO_2_ [81]. As a result, the depletion layer is formed at *n*-TiO_2_ surface due to loss of electrons, and accumulation layer is formed at SnO_2_ surface due to added electrons. In turn, an increase in the electron concentration stimulates the chemisorption of oxygen species enhancing the response formed due to reactions (1). The scheme in Figure 11a is constructed for the heterocontact SnO_2_ (cassiterite)/TiO_2_ (anatase), since the anatase phase is present in all nanocomposites (as it follows from the data of Raman spectroscopy). Brookite (revealed in SnTi-1 and SnTi-2 nanocomposites) is the least studied polymorph of TiO_2_. However, from the data of Ref. [82] it follows that the bottom of the conduction band of brookite lies above the bottom of the conduction band of anatase. Thus, in the formation of a heterocontact SnO_2_ (cassiterite)/TiO_2_ (brookite), electron transfer will also occur from TiO_2_ (brookite) to SnO_2_, increasing the chemisorption of oxygen on its surface. An increase in the concentration of chemisorbed oxygen on the surface of nanocomposites compared with unmodified SnO_2_ is confirmed by the method of thermo-programmed reduction with hydrogen (TPR-H_2_). Figure 11b shows the temperature dependencies of the hydrogen consumption during the reduction of unmodified SnO_2_ and SnTi-1 nanocomposite. The reduction of SnO_2_ with hydrogen occurs in the temperature range of 430–800 °C, the maximum H_2_ consumption is observed at T = 620 °C. The introduction of Ti-containing phases leads to a shift of the H_2_ consumption towards lower temperatures 570–600 °C. Such a change in the TPR-H_2_ profiles may be due to the reduction of highly dispersed Ti-containing phases. According to the reports [83,84,85], the reduction of TiO_2_ with hydrogen occurs at T > 400 °C, the maximum H_2_ consumption is observed at T = 530–650 °C, depending on the TiO_2_ dispersion. Another feature of the TPR-H_2_ profiles is the presence of hydrogen absorption peaks in the low-temperature range of 100–400 °C. This corresponds to the interaction of hydrogen with oxygen-containing particles (chemisorbed oxygen, hydroxyl groups, etc.) on the surface of highly dispersed semiconductor oxides. The reduction of TiO_2_@SnO_2_ nanocomposites is accompanied by more significant hydrogen consumption in the low-temperature region that indicates a higher concentration of oxygen-containing species on its surface. An increase in the concentration of chemisorbed oxygen on the surface of nanocomposites provides an increase in their resistance compared to unmodified SnO_2_. At the same time, due to the photodesorption of chemisorbed oxygen under UV light, TiO_2_@SnO_2_ nanocomposites are characterized by the larger photoresponse in the background air and demonstrate a more significant decrease in the sensor response to HCHO.

Thus, we are forced to conclude that in the case of TiO_2_@SnO_2_ nanocomposites, the amplitude of the sensor response when detecting HCHO in the sub-ppm range is determined mainly by the SnO_2_/TiO_2_ interface. The role of UV light is to enhance the photodesorption of oxygen, and the processes of HCHO oxidation by photoactivated particles (reactions (6) and (8)) do not contribute to the sensor response.

**Figure 12:**
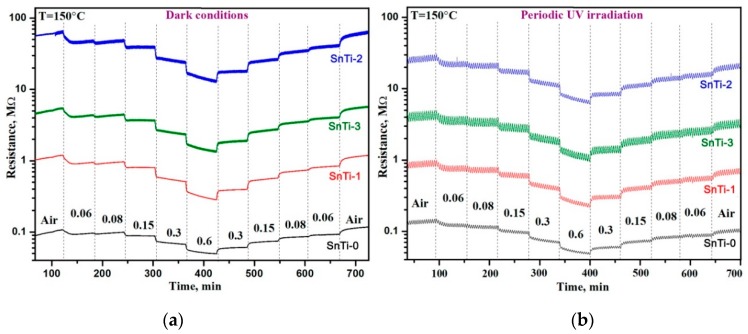
Resistance of the TiO_2_@SnO_2_ nanocomposites at T = 150 °C under stepwise change of the HCHO concentration (**a**) in dark conditions; (**b**) under periodic UV (λ_max_ = 365 nm) illumination. The numbers in the figure correspond to HCHO concentration (ppm).

**Figure 13:**
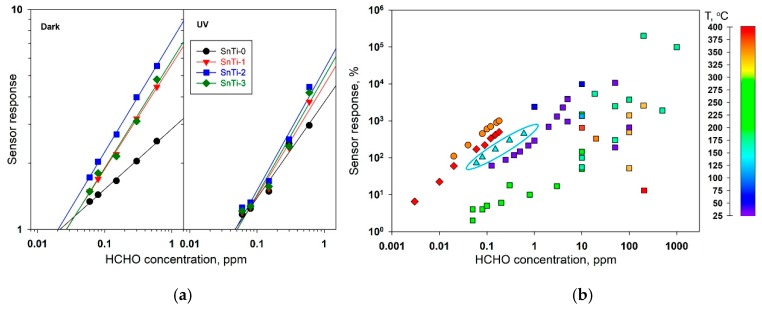
(**a**) Calibration curves for the HCHO detection using TiO_2_@SnO_2_ nanocomposites at T = 150 °C under stepwise change of the HCHO concentration in dark conditions (left) and under UV periodic illumination (right). (**b**) Comparison of the values of the sensor response of SnTi-2 nanocomposite (triangles) with the literature data [8,9,10,11,20,21,22,23,28,87,88,89,90,91,92,93,94,95,96,97,98,99,100,101,102,103,104,105,106,107,108,109,110,111,112,113,114,115,116,117] (squares), Ref. [7] (diamonds) and Ref. [86] (circles). The color of the symbol corresponds to the measurement temperature. For correct comparison all the data were recalculated as *S* = (*R_air_*/*R_gas_* - 1)*100%.

The dependence of the sensor response on the HCHO concentration in air was studied in dark conditions and under periodic UV illumination at 150 °C during the stepwise increase and subsequent stepwise decrease in the HCHO concentration (Figure 12). The results obtained allowed us to build the calibration curves (Figure 13a) and to determine the lower detection limit (LDL). As in the case of measurements at different temperatures, when using UV illumination, a decrease in the sensor response toward HCHO is observed, that may be due to the photodesorption of chemisorbed oxygen under UV light. The LDL values were calculated from the calibration curves as the gas concentration corresponding to the minimum measurable sensor signal R_av_/(R_av_ – 3σ) , where R_av_ is the average sensor resistance in air at 150 °C, σ is the standard deviation of the resistance in air. A decrease in the sensitivity of nanocomposites is accompanied by an increase in LDL from 9–15 to 30–32 ppb (Table 4).

The comparison of the sensor response toward 0.06–0.6 ppm HCHO for SnTi-2 nanocomposite with the literature data [7,8,9,10,11,20,21,22,23,28,86,87,88,89,90,91,92,93,94,95,96,97,98,99,100,101,102,103,104,105,106,107,108,109,110,111,112,113,114,115,116,117] is shown in Figure 13b and Table 5. There are very few papers that consider the detection of formaldehyde in a practically important sub-ppm concentration range. TiO_2_@SnO_2_ nanocomposites exhibit high sensitivity to HCHO in the sub-ppm range. Higher sensor response values were obtained only by the authors of [86], where metastable In_4_Sn_3_O_12_ was used as sensitive material at 350 °C and in Ref. [7], where Si modified SnO_2_ was used at 400 °C. It should be noted that TiO_2_@SnO_2_ nanocomposites have comparable sensitivity at much lower measurement temperature (150 °C vs. 350 °C or 400 °C), which can provide a significant reduction in the power consumption of semiconductor gas sensor.

**Figure 14:**
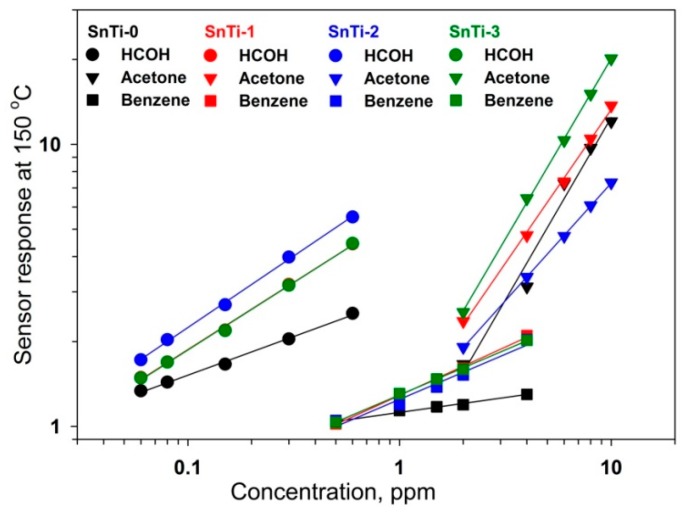
Cross sensitivity of SnO_2_ (SnTi-0, black), SnTi-1 (red), SnTi-2 (blue) and SnTi-3 (green) nanocomposites toward HCHO (circles), benzene (squares) and acetone (triangles) at 150 °C.

In addition to reducing power consumption, a sufficiently low measurement temperature allows one to achieve an increase in the selectivity of sensors when detecting various VOCs. The cross sensitivity of SnO_2_ and TiO_2_@SnO_2_ nanocomposites was studied at a measurement temperature of 150 °C in the detection of formaldehyde, benzene, and acetone (Figure 14). It can be noted that the synthesized materials have low cross-sensitivity to benzene in the wide concentration range. As for acetone, the interference of signals can occur if acetone concentration is in the range of C > 1 ppm and at least 20 times higher than formaldehyde concentration. SnTi-2 nanocomposite is characterized by the lowest response to acetone.

Comparing the sensor characteristics of unmodified SnO_2_ and TiO_2_@SnO_2_ nanocomposites in the detection of formaldehyde at 150 °C (Table 4) we can conclude that the SnO_2_ is inferior in the magnitude of the sensor response and the dynamic characteristics (response and recovery time). For HCHO detection at concentrations of 60 ppb and above, the SnTi-2 nanocomposite, which exhibits the highest sensor response and the lowest cross-sensitivity to acetone, seems to be preferred. However, for the measurements of lower HCHO concentrations, the SnTi-1 nanocomposite characterized by lower LDL value, lower base resistance in air, and the same dynamic characteristics, may be optimal.

As a summary, we have to conclude, that compared to the sensor characteristics described in [7,86,118], the sensors based on TiO_2_@SnO_2_ nanocomposites are inferior in sensitivity and selectivity. However, their advantage is significantly reduced operating temperature. The increase in sensitivity while maintaining a low operating temperature should be possible due to the addition of modifiers of different chemical nature on the surface of TiO_2_@SnO_2_ nanocomposites. The increase in selectivity, in particular towards humidity, can be achieved using passive filters—selective membranes based on SiO_2_ [6,118,119,120] or metal organic frameworks [110,121] as well as by the creation of multi-sensor systems operating using mathematical signal processing [6,122].

## 4. Conclusions

Nanocomposites TiO_2_@SnO_2_ obtained by ALD synthesis were investigated as sensitive materials for sub-ppm formaldehyde detection in dark conditions and under periodic UV (λ_max_ = 365 nm) illumination. As observed by XRD and Raman spectroscopy, all nanocomposites contain nanocrystalline SnO_2_ cassiterite and TiO_2_ anatase as the main crystalline phases. Depending on Ti content in nanocomposites predetermined by the number of ALD cycles, the minor TiO_2_ phases brookite, rutile and anosovite have been found. A thorough study of nanocomposites using FTIR and TPR-H_2_ methods made it possible to establish that nanocomposites have a higher concentration of chemisorbed oxygen and surface hydroxyl groups compared to unmodified SnO_2_. When detecting formaldehyde in the sub-ppm range, TiO_2_@SnO_2_ nanocomposites exhibit a higher sensor signal than SnO_2_ and a decrease in the optimal measurement temperature by 50 °C. This result is explained based on the model considering the formation of *n*-*n* heterocontact at the SnO_2_/TiO_2_ interface. TiO_2_@SnO_2_ nanocomposites have high sensitivity toward HCHO at quite low measurement temperature 150 °C that can provide a significant reduction in the power consumption and an increase in the selectivity of semiconductor gas sensors when detecting various VOCs.

Unexpectedly, UV illumination leads to a decrease in sensor response compared with the results obtained in dark conditions. So, we have to conclude that in the case of TiO_2_@SnO_2_ nanocomposites, the amplitude of the sensor response toward sub-ppm HCHO concentrations is determined mainly by the SnO_2_/TiO_2_ interface. The UV light stimulates the photodesorption of oxygen, while the processes of HCHO oxidation by photoactivated particles do not contribute to the sensor response.

## Figures and Tables

**Table 1:** Elemental and phase composition of SnO_2_ reference sample and TiO_2_@SnO_2_ nanocomposites with crystallite size *d_XRD_* of detected phases.

Sample	Number of ALD Cycles	[Ti]/([Ti]+[Sn]), mol.%	Phase Composition	
			Phases	*d_XRD_*, nm
SnTi-0	0	0	SnO_2_ cassiterite	9 ± 1
SnTi-1	500	20 ± 2	SnO_2_ cassiterite	5.1 ± 0.5
			TiO_2_ brookite	13 ± 1
			TiO_2_ anatase	n/d
SnTi-2	1000	24 ± 2	SnO_2_ cassiterite	4.5 ± 0.5
			TiO_2_ brookite	16 ± 2
			TiO_2_ anatase	n/d
SnTi-3	2000	39 ± 3	SnO_2_ cassiterite	3.5 ± 0.5
			TiO_2_ anatase	22 ± 2

**Table 2:** Assignment of Raman vibrational modes (cm^−1^).

SnTi-0	SnTi-1	SnTi-2	SnTi-3
137 (B_1g,_ cassiterite)	145 (E_g_, anatase)	145 (E_g_, anatase)	145 (E_g_, anatase)
309 (IR E_u_, cassiterite)	198 (E_g_, anatase)	198 (E_g_, anatase)	198 (E_g_, anatase)
352 (IR E_u_, cassiterite)	255 (A_1g_, brookite)	255 (A_1g_, brookite)	
444 (IR B_1u_, cassiterite)	285 (B_3g_, brookite)	285 (B_3g_, brookite)	
481 (E_g_, cassiterite)	397 (B_1g_, anatase)	397 (B_1g_, anatase)	397 (B_1g_, anatase)
563 (surface mode)	443 (B_3g_, brookite)	443 (B_3g_, brookite)	
631 (A_1g_, cassiterite)	516 (A_1g_+B_1g_, anatase)	516 (A_1g_+B_1g_, anatase)	516 (A_1g_+B_1g_, anatase)
773 (B_2g_, cassiterite)	581 (B_2g_, brookite)	581 (B_2g_, brookite)	
	635 (E_g_, anatase)	635 (E_g_, anatase)	635 (E_g_, anatase)

**Table 3:** Assignment of IR bands (cm^−1^) for the intermediates of HCHO oxidation.

Sample	Formaldehyde HCHO	DOM H_2_COO^-^	Formate HCOO^-^	Other Bands
SnTi-0	1202 τ (CH_2_)	1050 ν(CO)	2838 ν(CH)	2337 CO_2_
	1742 ν(CO)	1107 ρ(CH_2_)	2890 ν(CH)	2365 CO_2_
	1766 ν(CO)	1145 ρ(CH_2_)		
	3010	2936 δ (CH_2_)		
SnTi-2	1768 ν(CO)	1066 ν(CO)	1344 ν_s_(COO)	2340 CO_2_
		1107 ρ(CH_2_)	1346 ν_s_(COO)	3550 ν(OH)
		1146 ρ(CH_2_)	1382 δ(CH)	3630 ν(OH)
		1165 ρ(CH_2_)	1553 ν_ass_(COO)	
		1450 δ_s_(CH_2_)	1592 ν_ass_(COO)	
		1478 δ_s_ (CH_2_)	2590	
		2930 δ (CH_2_)	2868 ν(CH)	

**Table 4:** Sensor characteristics of unmodified SnO_2_ and TiO_2_@SnO_2_ nanocomposites in HCHO detection at 150 °C.

Sample	S (to 0.06 ppm)		LDL, ppb		R_av_, Ohm		τ_90_, s	τ*_90_, s
	Dark	UV	Dark	UV	Dark	UV		
**SnTi-0**	**1.34**	1.17	9	30	1.2·10^5^	1.1·10^5^	360	780
SnTi-1	1.49	1.20	9	32	1.2·10^6^	7.4·10^5^	120	660
SnTi-2	1.72	1.26	15	32	6.5·10^7^	2.2·10^7^	120	660
SnTi-3	1.48	1.21	10	30	5.8·10^6^	3.5·10^6^	120	680

**Table 5:** Literature data on sensor response *S* = (*R_air_*/*R_gas_* - 1)*100% of metal oxide semiconductor gas sensor in formaldehyde detection.

Material	HCHO conc., ppm	Response S	T, ^o^C	Ref.
Si-SnO_2_ FSP *^a^* films	0.003	6.5	400	[7]
In_4_Sn_3_O_12_ thin films	0.02	110	350	[86]
CuO nanocubes	0.05	4	300	[87]
SnO_2_-Au	0.05	2	300	[8]
In_2_O_3_ nanolamellas	0.08	4	300	[88]
TiO_2_ UV activation	0.1	67	RT	[22]
SnO_2_-NiO	0.3	18	300	[89]
Ag-LaFeO_3_	0.5	2400	40	[90]
Ag-LaFeO_3_	1	2400	90	[91]
TiO_2_ hollow microspheres	5	3900	RT	[23]
Au@ZnO core-shell structure	5	957	RT	[92]
CdO-In_2_O_3_	10	9900	95	[93]
SnO_2_ mesoporous	10	9900	150	[94]
TiO_2_ macroporous	10	6900	RT	[20]
Cd-In_2_O_3_ hollow fibers	10	1500	280	[95]
Ag-SnO_2_ nanoparticles	10	1340	125	[96]
SnO_2_/In_2_O_3_ nanofibers	10	650	375	[97]
SnO_2_ nanowires	10	145	270	[98]
SnO_2_/α-Fe_2_O_3_ hollow spheres	10	100	250	[99]
Pd-SnO_2_ thin films	10	55	250	[100]
NiO thin films	10	50	300	[101]
TiO_2_ nanotube arrays	10	7	RT	[21]
La_2_O_3_-SnO_2_ thin films	18.7	5400	250	[102]
NiO thin films	20	330	340	[103]
ZnO UV activated	50	10700	RT	[104]
TiO_2_-SnO_2_	50	3000	360	[28]
La_1-x_Sr_x_FeO_3_	50	2500	200	[105]
SnO_2_-MWCNT *^b^*	50	300	250	[106]
Au@SnO_2_ core-shell structure	50	190	RT	[107]
SnO_2_ microspheres	100	3730	200	[108]
Cd-TiO_2_/SnO_2_	100	1400	320	[109]
ZnO/ZIF *^c^*	100	1100	300	[110]
Au-ZnO octahedrons	100	660	RT	[111]
γ-Fe_2_O_3_	100	500	320	[77]
α-Fe_2_O_3_	100	52	325	[112]
CdO/Sn-ZnO	200	200000	200	[113]
ZnO-MnO_2_	200	2600	320	[114]
Ga-ZnO	205	13	400	[115]
LaFeO_3_ hollow nanospheres	500	1900	260	[116]
ZnO	1000	99600	210	[117]

*^a^* Flame spray pyrolysis; *^b^* Multiwall carbon nanotubes; *^c^* Zeolitic Imidazolate Framework.
